# Unraveling the Inactivation Mechanisms of Human Adenovirus 2 in Sunlight Disinfection: Synergism between Direct and Indirect Pathways

**DOI:** 10.1021/acs.est.5c06149

**Published:** 2025-09-18

**Authors:** Sujin Shin, Yunho Lee, Tamar Kohn

**Affiliations:** † Department of Environment and Energy Engineering, 65419 Gwangju Institute of Science and Technology (GIST), Gwangju, 61005, Republic of Korea; ‡ Laboratory of Environmental Virology, School of Architecture, Civil and Environmental Engineering, Swiss Federal Institute of Technology in Lausanne (EPFL), Lausanne 1015, Switzerland

**Keywords:** Sunlight disinfection, Human adenovirus, Synergism, Genome repair, Repair-deficient cells

## Abstract

Adenovirus (HAdV), a double-stranded DNA virus, exhibits resistance to direct inactivation by UVC and solar UVB radiation in water, owing to its ability to repair UV-induced genome damage using host cellular machinery. In the presence of dissolved organic matter (DOM), however, previous studies have reported unexpectedly rapid sunlight-mediated inactivation, even after accounting for indirect effects mediated by photochemically produced reactive intermediates (PPRIs). Here, we hypothesized that a synergistic interaction between direct and indirect inactivation pathways compromises HAdV2’s genome repair capacity, thereby enhancing its overall susceptibility to inactivation. First, we demonstrated that pre-exposure to singlet oxygen (^1^O_2_)—a key PPRI—significantly increased HAdV2’s susceptibility to subsequent direct UV inactivation. Using host cells with differing genome repair capacities, we further showed that simultaneous exposure to direct and indirect inactivation pathways reduced HAdV2’s genome repair efficiency compared to direct UV alone. Finally, although indirect inactivation caused minimal DNA damage, it impaired the DNA replication efficiency of host cells, likely due to oxidative damage to viral proteins involved in transcription-coupled repair. These findings highlight a critical interplay between direct and indirect inactivation pathways and offer new insights that can aid in optimizing light-based disinfection strategies to enhance the efficacy of water treatment processes targeting adenovirus-contaminated sources.

## Graphical abstract

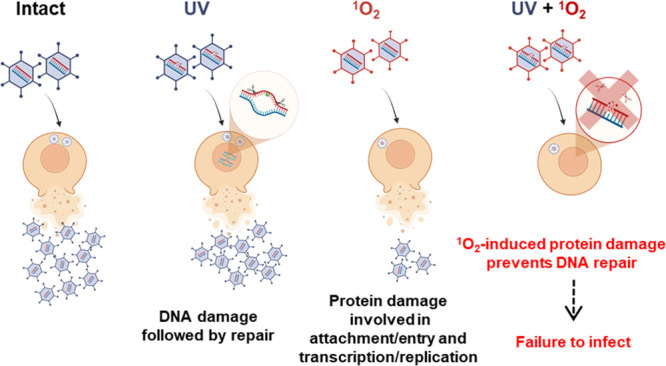

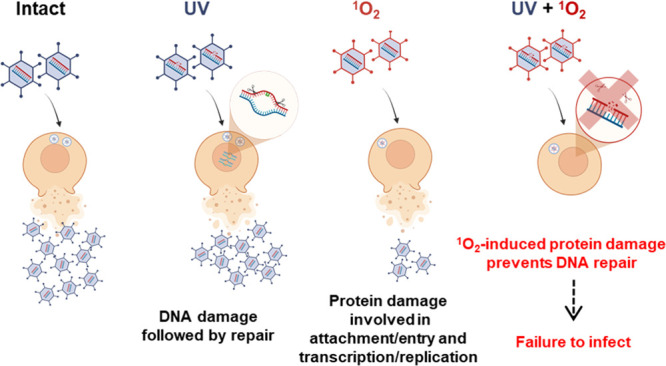

## 1 Introduction

Human adenoviruses (HAdVs) are ubiquitous pathogens that pose significant public health risks in both industrialized and developing countries, causing a wide range of diseases.^1^ Due to their widespread prevalence and potential health impacts, the United States Environmental Protection Agency (USEPA) has included HAdVs in the Contaminant Candidate List 4 (CCL4), highlighting them as a priority for future regulation in drinking water.^2^ HAdVs are transmitted through human-to-human contact and via contaminated drinking water, food and recreational water. They are frequently detected in wastewater effluents worldwide, with reported concentrations ranging from 10^1^ to 10^6^ genome copies per liter.^3–10^ A recent review reported that HAdVs were present in 52.6% of global surface water samples used as drinking water sources and 16.0% of drinking water samples.^11^ Among the various serotypes, HAdV2 is one of the most frequently isolated from natural water sources,^12^ and primarily affects young children with immature immune systems.^13^

In natural surface water systems, human pathogens can be efficiently inactivated by sunlight. Sunlight disinfection is a complex process involving multiwavelength UV light and various photochemically produced reactive intermediates (PPRIs) such as hydroxyl radical (^•^OH), singlet oxygen (^1^O_2_), and triplet states of dissolved organic matter (^3^DOM*). These PPRIs are produced via the interaction of sunlight with light-absorbing sensitizers (mainly DOM) present in natural waters. The wavelengths of sunlight that reach the surface of natural waters span the UVB (280–320 nm), UVA (320–400 nm), and visible light ranges.^14^ Working in sensitizer-free solutions, Beck et al. (2014, 2017)^15,16^ found that monochromatic light in the long UVC/UVB range (261, 268, 270, 278, 280, and 290 nm) caused minimal damage to viral proteins. These findings suggest that solar UVB inactivates HAdV primarily through damage to the viral DNA, a mechanism commonly referred to as direct inactivation. In contrast, PPRIs such as ^1^O_2_ have been reported to inactivate HAdV predominantly via capsid protein damage.^17^ According to Bosshard et al.,^17^ ^1^O_2_-induced capsid protein damage interferes with host cell attachment and entry, thereby contributing to HAdV inactivation. This mode of virus inactivation mediated by PPRIs is generally termed indirect inactivation.

To predict viral inactivation rates in sunlit surface water, Mattle et al.^19^ developed a model based on the quantum yield of UV-induced virus inactivation (i.e., direct inactivation) and second-order rate constants describing reactions between PPRIs and virus (i.e., indirect inactivation). The model assumes that direct and indirect inactivation pathways are additive, and under this assumption, it accurately predicted the inactivation rates of bacteriophages MS2 and phiX174. In contrast, the model underestimated the inactivation rate of HAdV2 by a factor 6 to 7. The reason for this discrepancy remains unclear, but may stem from an unrecognized synergistic interaction between direct and indirect inactivation pathways for HAdV.^19^

As one of the few double-stranded DNA (dsDNA) enteric viruses, HAdV possesses the ability to utilize host cellular machinery to repair UV-induced DNA damage. This repair mechanism renders HAdV more resistant to direct UV inactivation compared to viruses with other genome types.^20–26^ However, the efficacy of host-mediated repair for HAdV under solar disinfection conditions — where both direct and indirect inactivation processes occur simultaneously — remains poorly understood. In this study, we hypothesize that PPRIs impair the virus’s ability to undergo DNA repair, thereby making HAdV unexpectedly sensitive to solar inactivation, beyond what is expected from direct and indirect effects alone.

To test this hypothesis, we compared the direct UV inactivation kinetics of HAdV2 with those of HAdV2 pre-exposed to ^1^O_2_, a key PPRI. Additionally, we evaluated the effect of host cell DNA repair on HAdV2 inactivation rates by employing cell lines with differing DNA repair capacities. Finally, to elucidate the mechanisms underlying indirect inactivation, we monitored viral genome dynamics in infected host cells. The results are discussed in the context of HAdV fate in natural aquatic environments and potential strategies to improve the efficacy of light-based water disinfection processes.

## 2 Materials and Methods

### 2.1 Standards and Reagents

The chemicals used in this study are listed in SI-Text-1.

### 2.2 Virus Propagation, Cell Culture, and Enumeration

HAdV2 and A549 cells were kindly provided by Rosina Girones (University of Barcelona). HAdV2 was propagated using A549 cells. XP17BE cells were purchased from the American Type Culture Collection (ATCC, Manassas, VA, USA). A549 is a human lung epithelial cell line (ATCC CCL-185), while XP17BE is a fibroblast cell line derived from a patient with xeroderma pigmentosum (ATCC CRL-1360). XP17BE cells exhibit only 30–60% of the nucleotide excision repair efficiency of normal cells.^27^ Virus enumeration was performed using either A549 cells or XP17BE cells, and concentrations are expressed as most probable number per mL (MPN/mL). Detailed methods for virus propagation, cell culture, and virus enumeration are provided in SI-Text-2.

### 2.3 Solar Inactivation Experiments

Experiments were conducted using a Sun 2000 solar simulator (Abet Technologies, Milford, CT, USA), operated primarily at 25 A. For the experiments involving (1) Rose Bengal ^1^O_2_ photosensitization (Section 2.4) and (2) comparison of HAdV2 direct inactivation and its genome damage rates, the simulator was operated at 50 A. The system was equipped with a 1 kW xenon arc lamp, an air mass (AM) 1.5 filter, and an atmospheric edge (AE) filter (all from Abet Technologies), providing a realistic representation of full-spectrum sunlight (SI Figure S1). All experiments were conducted in 10 mM phosphate buffer at pH 7.0. The initial concentration of HAdV2 was 10^6^–10^7^ MPN/mL, except in ^1^O_2_ pre-exposure experiments, which required a higher starting concentration of 10^8^ MPN/mL. Details of the solar reactor setup and sampling procedures are provided in SI-Text-3. The intensity of simulated sunlight in the 280–400 nm range was determined using *p*-nitroanisole/pyridine actinometry^28^ (SI-Text-4), yielding an intensity of 1.4 × 10^–8^ Einstein/(cm^2^×s).

### 2.4 Rose Bengal ^1^O_2_ Photosensitization Experiment

Rose Bengal (RB, Fisher Scientific, Hampton, NH, USA) was used as a photosensitizer to predominantly generate ^1^O_2_ in experiments designed to investigate indirect inactivation mechanisms. Sunlight simulator intensity for RB photosensitization experiment was set to be 50 A. Samples were placed under glass Petri dishes to block UVB radiation (SI Figure S1). Control experiments conducted without RB under the same conditions yielded a first-order HAdV inactivation rate constant of approximately 0.12 h^–1^ (calculated based on ln­[C/C_0_] vs time; SI Figure S2), which was less than 1% of the rate constant observed in the presence of 10 μM RB (20 h^–1^). These results confirmed that ^1^O_2_ was the dominant player responsible for viral inactivation in the RB photosensitization system. Under simulated solar irradiation, RB underwent photobleaching, with 50% and 80% degradation observed after 2 and 4 h of exposure, respectively (SI Figure S3a). To maintain consistent RB levels during longer experiments (0.5 – 4 h), such as those measuring DNA degradation (see Section 2.8), RB was periodically replenished. Specifically, 250 μL of 1 mM RB stock solution was added to the reaction mixture (50 mL) every 2 h. Using this approach, the RB concentrations remained within 15% of the target 10 μM (data not shown). The steady state concentration of ^1^O_2_ measured in this system was 3.4 (±0.1)×10^–11^ M (SI Figure S3b).

### 2.5 Sequential Exposure to ^1^O_2_ and Solar UVB

HAdV2 samples (C_0_ = 10^8^ MPN/mL) were prepared in pH 7 phosphate buffer and spiked with 10 μM RB. HAdV2 samples were exposed to simulated sunlight for 0–15 min, corresponding to ^1^O_2_ exposures ranging from 1.0 × 10^–8^ to 3.1 × 10^–8^ M × s. Following exposure, the samples were purified using 15 mL centrifugal membrane filter units (100-kDa cutoff, Millipore) with phosphate-buffered saline (PBS). The initial 50 mL of sample volume was concentrated to 10 mL in PBS, then diluted with 90 mL of phosphate buffer for subsequent full-spectrum sunlight exposure.

### 2.6 Prediction of Inactivation Rate Constants

The first-order overall inactivation rate constant for HAdV2 (*k*_overall,pred_) was predicted using the model described by Mattle et al.^19^ This model assumes that direct and indirect photoinactivation pathways contribute independently and additively to the overall inactivation process. The indirect pathway includes contributions from PPRIs such as ^•^OH, ^1^O_2_, and ^3^DOM*, as expressed in eq 1.

1 koverall,pred=kdirect+k•OH[O•H]ss+k1O2[O12]ss+k3DOM*[D3OM*]ss

where *k*_direct_ is the first-order direct inactivation rate constant, while *k*_•OH_, *k*_1O2_, and *k*_3DOM*_ are the second-order rate constants for HAdV2 inactivation by ^•^OH, ^1^O_2_, and ^3^DOM*, respectively. These second-order rate constants were obtained from Mattle et al.^19^ The steady-state concentrations of PPRIs ([^•^OH]_ss_, [^1^O_2_]_ss_, [^3^DOM*]_ss,_) were experimentally determined in this study using appropriate probe compounds (SI-Text-5). The direct inactivation rate constant in DOM-containing solutions (*k*_direct_) was estimated using eqs 2 and 3, incorporating a light screening correction factor (CF) to account for photon attenuation by DOM:^29^

2 $$CF=\frac{\overset{400nm}{\underset{280nm}{\sum }}S_{\lambda }\times E_{p,\lambda }^{0}}{\overset{400nm}{\underset{280nm}{\sum }}E_{p,\lambda }^{0}}$$

Here, *E*_*p*,λ_^0^ is the spectral photon irradiance at wavelength λ [Einstein/(cm^2^×s)] (SI-Text-4), and *S*_λ_ is the wavelength-specific screening factor, calculated as *S*_λ_ = $\frac{1-10^{-\alpha_{\lambda }z}}{2.303\alpha_{\lambda }z}$ , where, α_λ_ is the decadic absorbance of DOM at wavelength λ, and z (cm) is the optical path length of the reactor. The calculated CF values were 0.79 and 0.67 for Suwanee River Natural Organic Matter (SRNOM) concentrations of 10 and 15 mgC/L, respectively.

The CF-corrected direct inactivation rate constant was then obtained as

3 $$k_{\mathrm{direct}}=\mathrm{CF}\times k_{\mathrm{direct},\mathrm{buffer}}$$

where *k*_direct,buffer_ is the first-order rate constant determined in phosphate buffer without DOM (from linear regression of ln­(C/C_0_) vs irradiation time). All virus inactivation kinetics were modeled as first-order with respect to irradiation time, and reported as *k*_obs_.

### 2.7 Viral Genome Monitoring

Quantification of viral genome copies from infected host cells was conducted using qPCR for the following purposes: (1) to assess the efficiency of virus attachment and entry into host cells, and (2) to evaluate the virus genome transcription and replication efficiency. This method was adapted from the approach developed by Gall et al.^30^ In the present study, a solution containing 10^6^ MPN/mL of HAdV2 was pretreated with a ^1^O_2_ exposure of 3.1 × 10^–8^ M × s, which resulted in approximately a 2-log_10_ inactivation. The virus solution was then diluted 1,000-fold into DMEM supplemented with 2% FBS, and 2 mL of this diluted solution was inoculated onto A549 monolayers prepared to be over 90% confluent in a 6-well plate and preincubated at 4 °C for 1 h. Cells infected with untreated virus (time zero) served as experimental controls. The inoculated cells were incubated with HAdV2 for 90 min at 4 °C in the dark to allow for virus binding to host cells while preventing virus entry and genome replication.^30^ Following incubation at 4 °C, the inoculum was removed, and monolayers were washed twice with 2 mL of ice-cold PBS to remove unbound viruses. Afterward, 2 mL of DMEM supplemented with 2% FBS was added, and the cells were transferred to a 37 °C CO_2_ incubator — this time point was considered as the start of infection.

At the designated time points after incubation, cells were trypsinized and subjected to three freeze–thaw cycles to release virions.^31^ The resulting supernatant containing HAdV2 was collected, and viral DNA was extracted using the PureLink viral RNA/DNA mini kit (Invitrogen, cat. no. 12280-050). Viral genome was quantified using a nested long-range PCR followed by qPCR (LR-PCR - qPCR), targeting a 1.1-kbp region of the hexon gene as a representative marker of the full viral genome (SI-Text-6). To normalize viral genome levels across samples, the human β-actin gene — a stably expressed housekeeping gene — was used as an internal control, enabling correction for differences in cell recovery number.^30,32^ Primer sets used for amplification are listed in SI Table S1. For qPCR analysis, the standard curves for the 1.1 kbp LR-PCR products of the HAdV2 hexon gene and the 103 bp human β-actin gene showed efficiencies of 105% and 91%, respectively (SI Figure S4).

### 2.8 Genome Damage Measurement

Viral DNA from HAdV2 exposed to either full-spectrum irradiation (50 A) or RB photosensitization (50 A, 10 μM RB in a covered glass Petri dish) was extracted using the PureLink viral RNA/DNA mini kit. Genome damage was assessed by quantifying degradation of the HAdV2 Hexon gene using a nested LR-PCR followed by qPCR, targeting a 1.1 kbp amplicon (see SI-Text-6 for details).

### 2.9 Statistical Analysis

All statistical analyses were performed using the R software (https://www.r-project.org/).

## 3 Results and Discussion

### 3.1 HAdV2 Inactivation by Sunlight Exceeds Additive Effects of Direct and Indirect Pathways

In a first step, we aimed to validate the finding of Mattle et al.,^19^ who reported that solar inactivation kinetics of HAdV2 exceeded the sum of direct and indirect inactivation pathways. To evaluate this, HAdV2 samples were exposed to simulated sunlight in the presence of SRNOM, and the observed inactivation rate constants (*k*_obs_) were compared to model predictions based on eq 1. SRNOM concentrations were set at 10 and 15 mgC/L, comparable to those used in the previous study.^19^ Experiments conducted in phosphate buffer without SRNOM were used to determine *k*_direct,buffer_, which was then used to estimate *k*_direct_ in eq 3. The steady-state concentrations of ^•^OH, ^1^O_2_, and ^3^DOM* in the presence of SRNOM were experimentally determined using specific probe compounds (SI-Text-5), and the corresponding values are summarized in SI Table S2.

In clear buffer, an HAdV2 inactivation rate constants *k*_obs_ of 0.10 h^–1^ was observed (Figure 1), and this value was used to predict the *k*_direct_ under SRNOM conditions at 10 and 15 mgC/L. In the absence of SRNOM, the *k*_overall,pred_ was equivalent to the measured *k*_obs_. In the presence of SRNOM, the *k*_obs_ increased to 0.17 and 0.21 h^–1^ under 10 and 15 mgC/L, respectively. However, according to the model prediction, *k*_direct_ decreased due to light screening by SRNOM, while the contribution of PPRIs increased, resulting in an *k*_overall,pred_ similar to that observed in the absence of SRNOM. The contribution of PPRIs was primarily attributed to singlet oxygen (^1^O_2_). Contributions from hydroxyl radicals (^•^OH) and triplet state DOM (^3^DOM*) were negligible (Figure 1 and SI Table S3). Under both 10 and 15 mgC/L SRNOM conditions, the *k*_obs_ exceeded the model predictions by factors of 1.5 and 1.7, respectively. Although the relative differences were smaller than those reported by Mattle et al. (6- to 7-fold), this consistent discrepancy suggests the involvement of an additional, unidentified mechanism that enhances HAdV2 inactivation beyond the additive contributions of direct and indirect processes. Control experiments conducted in the dark with 15 mgC/L SRNOM showed no significant change in viral concentration over time (SI Figure S5), confirming that observed effects were light-dependent.

**Figure 1:**
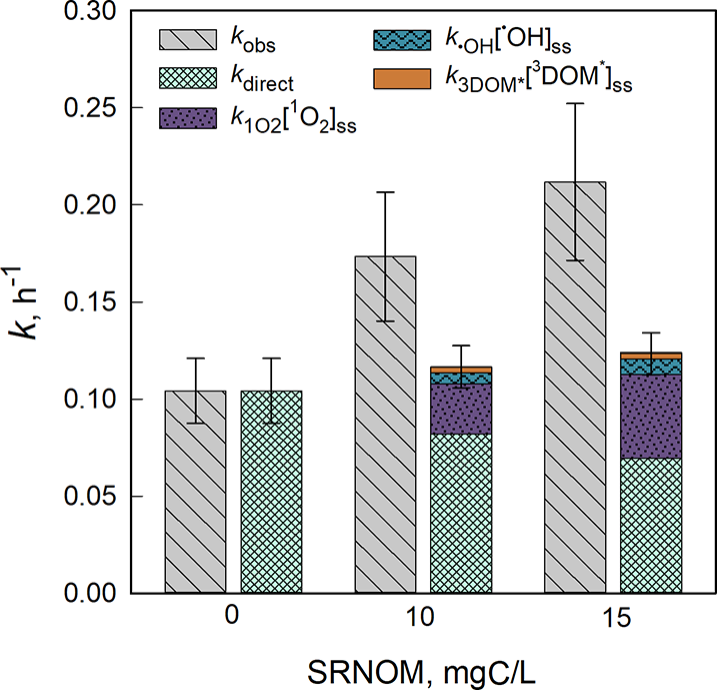
Measured (*k*_obs_, left with dashed gray) and predicted (*k*_overall,pred_, right) inactivation rate constants of HAdV2 in SRNOM-spiked buffer exposed to simulated sunlight. The checkered light green portion of the bar indicates the contribution of direct inactivation, calculated using eq 3. The dotted purple, wavy blue, and solid orange portions represent the contribution of singlet oxygen (^1^O_2_), hydroxyl radical (^•^OH), and triplet excited states of DOM (^3^DOM*), respectively, calculated based on second-order inactivation rate constants and the measured steady-state concentrations of the PPRIs (see SI Table S3). Error bars for *k*_obs_ reflect the standard error calculated from two pooled replicate experiments. For the *k*_overall,pred_, error bars represent propagated errors, primarily driven by the error in the direct inactivation rate.

### 3.2 Pre-exposure to ^1^O_2_ Enhances Subsequent UVB Inactivation of HAdV2

To investigate the mechanisms underlying the accelerated solar inactivation of HAdV2 in the presence of DOM, we examined whether the enhancement in *k*_obs_ could be attributed to a synergistic interaction between direct and indirect inactivation processes. To test this, the two pathways were temporally decoupled: HAdV2 was first exposed to ^1^O_2_, generated via RB photosensitization under UVB-filtered simulated sunlight, followed by subsequent exposure to full-spectrum simulated sunlight in the absence of PPRIs. Among the PPRIs, ^1^O_2_ was selected as the representative species for the PPRI-UV sequential experiment due to its substantial contribution to inactivation (Figure 1), as well as its moderate reactivity, reaction selectivity, and small molecular size, which may enable it to diffuse through the viral capsid and interact with internal components. In contrast, ^•^OH, due to its extremely high reactivity, is likely quenched at the capsid surface, while the relatively large molecular size of ^3^DOM* may hinder its access to internal viral targets.

The ^1^O_2_ exposure was varied between 1.0 × 10^–8^ and 3.1 × 10^–8^ M × s, corresponding to a maximum virus inactivation level of ∼ 2-log_10_ (99%). Following ^1^O_2_ exposure, the virus samples were washed to remove residual RB and then irradiated under full-spectrum simulated sunlight. A rapid drop in viral concentration was observed during the first hour of irradiation in all pre-exposed samples (SI Figure S6), likely due to trace amounts of residual RB. To minimize this potential confounding effect, *k*_obs_ was calculated using data from 1 to 4 h, after significant photobleaching of RB had occurred. During this period, the inactivation followed first-order kinetics (SI Figure S6).

Interestingly, HAdV2 samples pre-exposed to ^1^O_2_ at 1.0 × 10^–8^ M × s exhibited a 3.0-fold increase in *k*_obs_ compared to non-exposed samples (ANCOVA, p = 0.0003), confirming a synergistic interaction between the indirect and direct inactivation pathways (Figure 2). However, higher ^1^O_2_ exposures of 2.1 × 10^–8^ M × s and 3.1 × 10^–8^ M × s led to more modest enhancements in *k*_obs_ — 1.9-fold (p = 0.049) and 1.2-fold (p = 0.5913), respectively — suggesting a threshold beyond which further ^1^O_2_ exposure yields diminishing returns. While the mechanisms resulting in this nonmonotonic trend in *k*_obs_ with increasing ^1^O_2_ exposure are not investigated herein, this behavior may be linked to an effect of viral aggregation. Specifically, exposure to oxidants has been shown to cause aggregation of viral proteins^33^ or entire virions.^34^ Such aggregation could promote coinfection of cells and facilitate recombination, thereby reducing the overall UV inactivation efficiency, as suggested in Mattle and Kohn.^35^ In this context, viral aggregation at high ^1^O_2_ levels may counteract the enhanced UV susceptibility resulting from ^1^O_2_-induced protein damage. Our findings of a synergistic effect between oxidative (indirect) and UV inactivation align with those reported by Rattanakul et al.^36^ who observed enhanced UV inactivation of adenovirus following prechlorination. In their study, the synergistic effect was evident only when chlorination preceded UV exposure, but not the reverse. The authors proposed that damage to viral proteins may reduce infectivity and increase susceptibility to UV, although the precise mechanism has yet to be fully elucidated.

**Figure 2:**
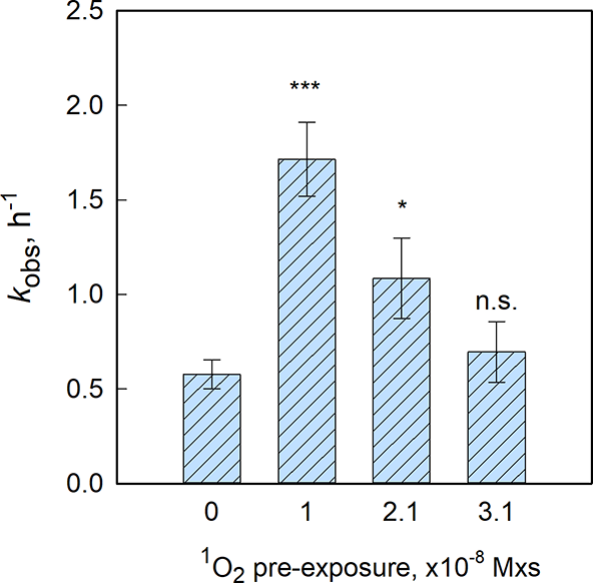
Measured inactivation kinetics of HAdV2 under simulated sunlight following different levels of ^1^O_2_ pre-exposures. For the control virus (no ^1^O_2_ pre-exposure), *k*_obs_ was determined using data from the 0–8 h range. For ^1^O_2_ pre-exposed samples, *k*_obs_ was determined from the 1–4 h range (SI Figure S6). Error bars represent the standard error associated with *k*_obs_, based on pooled duplicate experiments. An ANCOVA test was conducted to assess whether the first-order inactivation kinetics of ^1^O_2_ pre-exposed samples (1.0 × 10^–8^–3.1 × 10^–8^ M × s) differed significantly from the non-exposed control. Significance levels are indicated above the bars: ***­(*p* ≤ 0.001), *­(0.01 < *p* ≤ 0.05), and n.s. (not significant, *p* > 0.05).

### 3.3 Assessing Host DNA Repair Using Cell Lines with Differing Repair Capacities

Next, we tested the hypothesis that the accelerated HAdV2 inactivation kinetics observed in prior sections result from inhibition of the viral DNA repair process in host cell. To assess this, we compared HAdV2 inactivation in two cell lines with differing DNA repair capacities: XP17BE (30–60% DNA repair-efficient) and A549 (DNA repair-competent). Inactivation experiments were conducted under three different conditions: (i) dominated by direct inactivation (phosphate buffer, full-spectrum sunlight), (ii) dominated by indirect inactivation (10 μM RB, UVB-filtered sunlight), and (iii) mixed conditions involving SRNOM (10 or 15 mgC/L, full-spectrum sunlight), where direct and indirect inactivation occur simultaneously, but at varying proportions (Figure 1). Comparison of *k*_obs_ values measured in XP17BE and A549 cells allowed us to assess the extent of host-mediated DNA repair under each condition.

In the system dominated by direct inactivation, *k*_obs_ was 4.3-fold higher in XP17BE than in A549 cells (ANCOVA, p = 0.0015, Figure 3 **a and** Table 1), confirming that DNA repair in A549 cells greatly reduces HAdV2 susceptibility to direct UV damage. This result aligns with an earlier report showing that HAdV5 required 2.9 times higher UVC doses to achieve 4-log_10_ inactivation in HEK293 (DNA repair-competent) cells compared to XP17BE.^27^ In contrast, under the condition dominated by indirect inactivation, *k*_obs_ values in XP17BE and A549 cells were not significantly different (ANCOVA, p = 0.2670, Figure 3b and Table 1), indicating that host cell DNA repair plays no significant role in mitigating indirect damage induced by ^1^O_2_. This supports the notion that indirect inactivation is not primarily due to DNA damage, but rather aligns with the findings of Bosshard et al.^17^ who demonstrated that ^1^O_2_-driven inactivation of HAdV2 mainly targets viral proteins.

**Figure 3:**
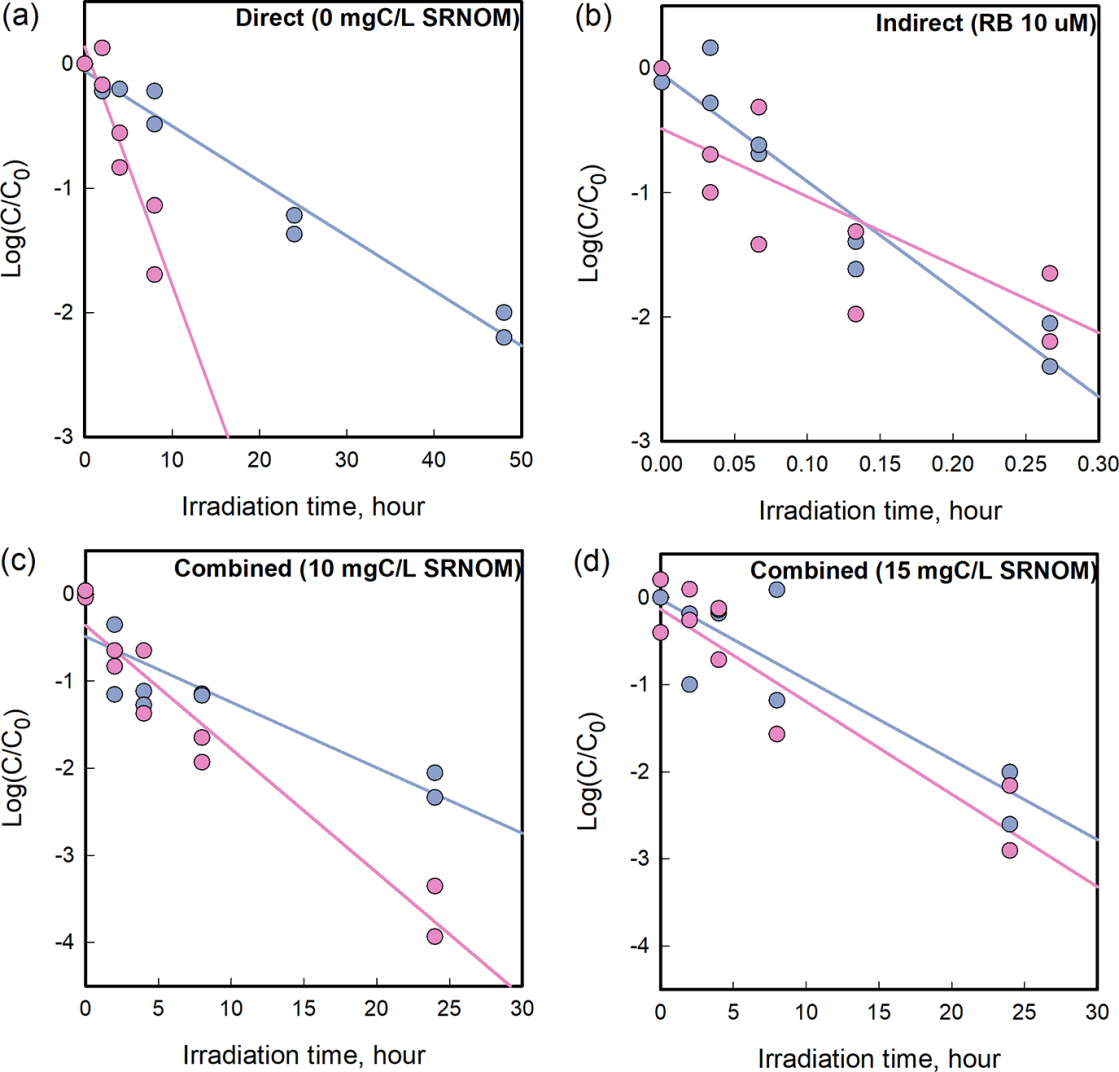
Measured inactivation kinetics of HAdV2 in A549 (dark pastel blue) and XP17BE (pink) under simulated sunlight. (a) Direct inactivation-dominant system (phosphate buffer, full-spectrum sunlight), (b) indirect inactivation-dominant system (10 μM RB, UVB-filtered sunlight), and (c and d) mixed systems where direct and indirect inactivation co-occur in the presence of SRNOM at 10 and 15 mgC/L, respectively, under the full-spectrum sunlight. All experiments were performed in pH 7 phosphate buffer (10 mM), with initial HAdV2 concentrations of 10^7^ MPN/mL. Each condition was tested in duplicate.

**Table 1:** Measured Inactivation Rate Constants of HAdV2 in Two Host Cell Lines (*k*_A549_ and *k*_XP17BE_ for A549 and XP17BE, Respectively) under Different Treatment Conditions^a^

	Direct SRNOM, 0 mgC/L	Indirect RB, 10 μM	Combined SRNOM, 10 mgC/L	Combined SRNOM, 15 mgC/L
*k*_A549_	0.10 (±0.04) h^–1^	20 (±4.8) h^–1^	0.17 (±0.08) h^–1^	0.21 (±0.09) h^–1^
*k*_XP17BE_	0.44 (±0.17) h^–1^	15 (±8.4) h^–1^	0.33 (±0.07) h^–1^	0.24 (±0.09) h^–1^
ANCOVA p-value	0.0015	0.2670	0.0033	0.5599
*k*_XP17BE_ **/** *k*_A549_	4.2 (±2.2)	0.8 (±0.5)	1.9 (±1.0)	1.2 (±0.8)

a Errors represent the 95% confidence intervals associated with *k*_obs_ and the XP17BE/A549 ratio.

In SRNOM-containing systems, where both direct and indirect inactivation pathways co-occur, the difference in *k*_obs_ between XP17BE and A549 cells corresponded to 1.9-fold and 1.2-fold at 10 and 15 mgC/L SRNOM, respectively (ANCOVA, p = 0.0033 and p = 0.5599, Figure 3c and 3d and Table 1). Compared to the direct inactivation system, this represents a 56% and 72% reduction in host DNA repair efficacy, respectively, despite only a 21% and 33% reduction in UV exposure due to light shielding by SRNOM (see section 2.6). This suggests that host-mediated DNA repair is effective when direct inactivation acts alone but becomes less efficient when direct and indirect pathways occur simultaneously, possibly due to interference by indirect damage with the cellular DNA repair machinery.

### 3.4 ^1^O_2_ Induced Viral Protein Damage Impairs Genome Replication

The findings thus far suggest that the indirect inactivation pathway involving ^1^O_2_ enhances UV inactivation of HAdV2 by inhibiting DNA repair. To gain a better understanding of ^1^O_2_-specific inactivation mechanisms of HAdV2, we monitored viral genome replication of ^1^O_2_ treated HAdV2.

The process of adenoviral infection in host cells involves multiple steps: attachment of the virus to the host cell via interaction between the HAdV2 fiber protein and the coxsackie-adenovirus receptor (CAR) of the host cell surface;^37,38^ endocytosis mediated by interaction between the penton base protein of HAdV2 and host cell integrins;^39^ translocation of the viral genome-protein VII complex, known as the Ad core, into the host cell nucleus;^40,41^ expression of the early E1A gene to establish a favorable environment for further replication;^42^ followed by genome replication and virion assembly. Monitoring viral genome copy numbers at different time points postincubation provides insight into the efficiency of each stage and how these may be impacted by different treatments.

According to prior studies,^30,32^ transcription of the early E1A gene begins approximately 1–2 h post incubation, while viral genome replication is initiated at 5–8 h. A full replication cycle is completed within 48 h, at which point newly assembled viral particles are released from the host cell to infect adjacent cells. In this study, hexon gene copy numbers were measured at 2 and 48 h post incubation (C_2h_ and C_48h_, respectively) to assess viral attachment/entry and replication efficiencies. For a virus input dose of 2 × 10^1^ to 2 × 10^3^ MPN per well, the genome copy number measured at 48 h was found to increase proportionally with the input dose (see SI-Text-7 and SI Figure S5). Based on these results, the viral input dose was maintained at or below 2 × 10^3^ MPN per well in all experiments.

The genome copy number of ^1^O_2_-treated HAdV2 at 2 h of incubation was 0.6-log_10_ lower than that of the untreated control, indicating a reduction in viral attachment and entry efficiency (Figure 4). The replication efficiency of successfully internalized genomes was evaluated by comparing the genome copy number at 48 and 2 h post incubation (C_48h_/C_2h_). In the untreated control, viral genome copies increased approximately 8,100-fold over this period, whereas ^1^O_2_-treated viruses showed only a 200-fold increase. This significant reduction in replication capacity is unlikely to result from extensive ^1^O_2_-induced DNA damage, as only minimal DNA damage was observed in the 1.1 kbp region amplified by LR-PCR, in contrast to direct inactivation (SI Figure S8). In other words, the viruses exposed to ^1^O_2_ exhibited a strong decrease in genome replication efficiency relative to untreated controls. This suggests that ^1^O_2_-mediated viral inactivation involves the disruption of DNA processing functions, such as transcription and replication, rather than genome degradation itself. Consequently, the ^1^O_2_-driven indirect inactivation appears to impair multiple protein functions, specifically host attachment and entry, as well as the virus’s ability to replicate its genome. This is consistent with previous observations for free available chlorine, which significantly hindered viral genome transcription and replication.^30^

**Figure 4:**
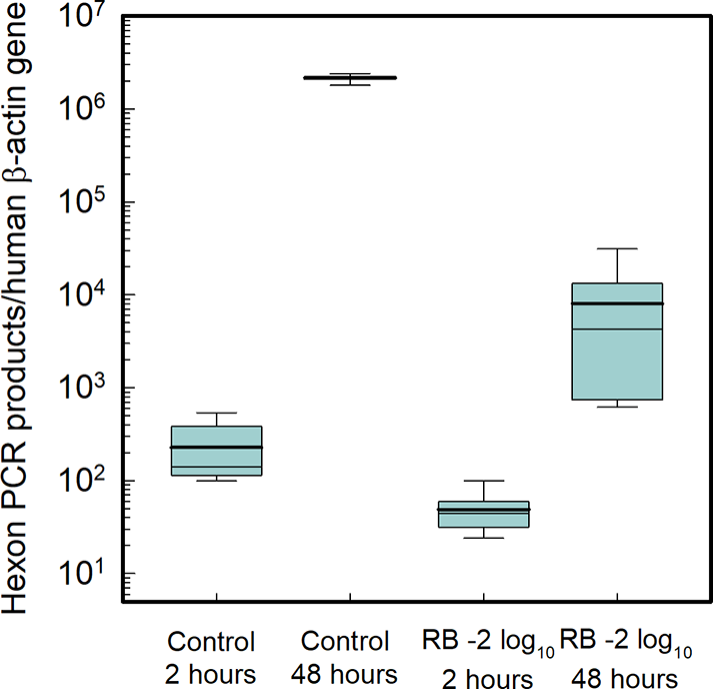
HAdV2 genome copies at 2 and 48 h of incubation. Viral genome copy numbers were normalized to human β-actin genome copies to account for variations in cell recovery efficiency across samples. The control represents untreated virus, while the -2 log_10_ sample refers to virus pretreated with ^1^O_2_ to achieve a 2-log_10_ reduction in infectivity. The thin line within each box indicates the median, and the thick line shows the mean. All experiments were conducted independently in triplicate.

One likely target involved in genome transcription and replication is protein VII (pVII), which binds the adenoviral genome to form the nucleoprotein ‘Ad core’ within the virion. According to Johnson et al.^43^ this core enters the nucleus and facilitates early E1A transcription prior to pVII dissociation. Notably, pVII contains amino acid residues such as methionine, histidine, tyrosine, and tryptophan (as identified in the ENA entry AAA92212.1 ^44^), which are known to react readily with ^1^O_2_, with reported second-order rate constants ranging from 7 × 10^6^ to 9 × 10^7^ M^–1^s^–1^.^45,46^ Given the small molecular size and sustained production of ^1^O_2_, it is plausible that it penetrates the virion capsid and reacts with pVII, thereby impairing its function in early viral replication. Taken together with earlier observations that ^1^O_2_ damage hinders the repair of UV-induced DNA lesions (section 3.3), this suggests that ^1^O_2_-induced damage to pVII — or to other essential replication-associated proteins — may disrupt transcription-coupled repair mechanisms and reduce efficient genome replication efficiency.

These findings help contextualize earlier observations. In the sequential ^1^O_2_-sunlight exposure experiments (Figure 2), viruses predamaged by ^1^O_2_may have experienced two outcomes: some failed to enter host cells, while others entered but were unable to effectively repair UV-induced genome damage. This combined impairment likely contributed to the observed increase in *k*_obs_ by reducing the number of viruses capable of completing successful genome replication.

A similar interpretation applies to the cell line comparison experiment (Figure 3), where the simultaneous action of direct and indirect pathways could have interfered with host-mediated DNA repair. This interference may account for the convergence of *k*_obs_ values observed at elevated DOM concentrations in DNA repair-competent and -deficient cell lines.

### 3.5 Implications

This study investigated the interdependence of direct and indirect solar inactivation pathways for HAdV2 and elucidated key mechanisms underlying their interaction. We demonstrate that PPRIs, particularly ^1^O_2_, enhance direct UV inactivation by impairing the repair of UV-induced genome damage, likely through oxidative modification of viral proteins involved in transcription-coupled genome repair and replication. These findings, while obtained for solar inactivation, also offer valuable insights for optimizing virus disinfection strategies, particularly in settings where enhanced viral inactivation is critical, such as in the treatment of hospital wastewater with high viral loads. Specifically, our results suggest that sequential or simultaneous application of chemical oxidants followed by UV irradiation may be more effective than UV treatment alone or than UV treatment followed by chemical oxidation. Chemical oxidants commonly used in UV-based advanced oxidation processes — such as chlorine — may contribute to enhanced HAdV UV inactivation by targeting nitrogen-containing residues in HAdV core proteins. However, before practical implementation, further testing is required to optimize oxidant exposure levels and assess potential impacts on treatment efficacy. Beyond engineered systems, our results also raise the possibility that a virus’s prior exposure to oxidative stress in natural environments may influence its susceptibility to subsequent UV-based inactivation. This highlights the importance of considering viral life history and environmental context in both natural and engineered disinfection scenarios.

## Supplementary Material



## Data Availability

All data discussed in this manuscript are accessible via https://doi.org/10.5281/zenodo.15354761.

## Notes

Standards and reagents used for experiments, virus propagation, cell culture, and virus enumeration methods, solar reactor setup and sampling procedures, actinometry using PNA/Pyr, quantification of PPRIs, nested LR-PCR and qPCR analysis methods, and determination of virus input dose for genome monitoring experiment (PDF)
