# Supplementary material for: Unraveling the Inactivation Mechanisms of Human Adenovirus 2 in Sunlight Disinfection: Synergism between Direct and Indirect Pathways
Source: Environ Sci Technol. 2025 Sep 18;59(38):20608–15. doi: 10.1021/acs.est.5c06149 (PMC12490006; doi:10.1021/acs.est.5c06149)
Supplement: Supplementary file 1 [file es5c06149_si_001.pdf]

Supporting information for

Unraveling the inactivation mechanisms of human adenovirus 2 in sunlight disinfection: Synergism between direct and indirect pathways

Sujin Shin [a,b], Yunho Lee [a,*] and Tamar Kohn [b,*]

[a] Department of Environment and Energy Engineering, Gwangju Institute of Science and Technology (GIST), Gwangju, 61005, Republic of Korea

[b] Laboratory of Environmental Virology, School of Architecture, Civil and Environmental Engineering, Swiss Federal Institute of Technology in Lausanne (EPFL), Lausanne, 1015, Switzerland

*Corresponding author. Mailing address: Department of Environment and Energy Engineering, Gwangju Institute of Science and Technology (GIST), Gwangju, 61005, Korea.

Phone: 82-62-715-2468, fax: 82-62-715-2434, email: yhlee42@gist.ac.kr

*Corresponding author. Mailing address: School of Architecture, Civil and Environmental Engineering, Swiss Federal Institute of Technology in Lausanne (EPFL), Lausanne, 1015, Switzerland.

Phone: 41-21-693-0891, fax: 41-21-693-80-70, email: tamar.kohn@epfl.ch

This SI includes 7 texts, 8 figures, 3 tables as supplementary materials, data, and discussions.

Supporting Texts

SI-Text-1. Standards and reagents.

The following chemicals were purchased from various suppliers and used without further purification: acetonitrile (A998SK, Fisher), ethanol (083136, OCI), furfuryl alcohol (FFA, 98-00-0, Sigma), hydrochloric acid (HCl, 37%, 7647-01-0, Merck), 2-hydroxyl terephthalic acid (2-HTA, 636-94-2, Sigma), methanol (A452SK, Fisher), *p*-nitroanisole (PNA, 100-17-4, Sigma), phosphoric acid solution (W290017, Sigma), pyridine (Pyr, 110-86-1, Sigma), rose bengal (RB, 85%, 10730891, Fisher), sodium phosphate dibasic dodecahydrate (98.5%, 271060025, Fisher), sodium phosphate monobasic dihydrate (99+ %, 271750010, Fisher), terephthalic acid (TA, 100-21-0, Sigma), and 2,4,6-trimethylphenol (TMP, 527-60-6, Sigma).

Stock solutions were prepared with ultrapure water (≥18.2 MΩ-cm), obtained using a Milli-Q® Advantage A10 purification system (Millipore, USA). Stock solutions of FFA, RB, and TMP (1 mM in ultrapure water) and Suwannee River natural organic matter (SRNOM, 700-800 mgC/L in phosphate buffer, filtered through a 0.45 μm filter) were prepared and stored at 4 °C until use.

SI-Text-2. Virus propagation, cells culture, and enumeration

Virus propagation. A549 cells were cultivated in 75-cm^2 culture flasks until reaching 95% confluence. The culture medium was then replaced with Dulbecco's modified Eagle's medium (DMEM) supplemented with 2% fetal bovine serum (FBS; Gibco, Thermo, Waltham, MA, USA) and 1% of penicillin-streptomycin (P/S; Gibco, Thermo, Waltham, MA, USA), followed by inoculation with HAdV2 at a concentration of 10^8 – 10^9 MPN/mL. Cells were incubated until cytopathic effects (CPE) were observed, characterized by cell death and detachment of approximately 80% of the cell monolayer. After 3–5 days, cells underwent three freeze-thaw cycles to lyse any remaining infected cells [1]. The resulting lysate was centrifuged at 13,000 × g to remove cellular debris, and the supernatant

was collected. Viruses were then concentrated using 15-mL centrifugal membrane filter units with a 100-kDa cutoff (Millipore). The final HAdV2 stock (10^{11} MPN/mL) was aliquoted and stored at -80 °C until use.

Cell culture. A549 and XP17BE cells were cultured in DMEM supplemented with 10% FBS, and maintained in 75-cm² culture flasks at 37°C in a humidified incubator with 5% CO_2. Subculturing was performed at a 1:8 dilution ratio for A549 cells and a 1:3 dilution ratio for XP17BE cells when confluence reached 70–80%.

Virus enumeration. Concentrations of infectious viruses were determined using a most probable number (MPN) assay in 96-well plates seeded with A549 or XP17BE cells. Samples were serially diluted 10-fold in DMEM supplemented with 2% FBS, and 200 μL of each dilution was inoculated onto monolayers in five replicate wells per dilution. Plates were incubated at 37°C with 5% CO_2 for 7–10 days, and CPE was assessed using inverted microscopy. The number of CPE-positive wells at each dilution was recorded, and quantified according to the MPN method [2] using the R package {MPN}. The lower limit of detection (LOD) was defined as the concentration at which CPE one out of five wells exhibited CPE at the lowest dilution, corresponding to 9.04 MPN/mL in this study.

SI-Text-3. Solar reactor setup and sampling procedures

The irradiance of the simulator was measured using an ILT 900-R spectroradiometer (International Light Technologies). The sunlight irradiance (μW/cm²) measured by a radiometer (**SI Figure S1a**) was converted into photon irradiance and integrated from 280 to 400 nm to result of 0.8×10^{-8} Einstein/cm²/s. Unless otherwise specified, all experiments were conducted under full-spectrum simulated sunlight. Sterilized, black-painted open glass beakers (50 mL) containing virus solutions were covered with UV-transmissible plastic wraps to minimize evaporation and prevent external contamination. A comparison of spectral irradiance between wrapped and unwrapped samples showed

no significant differences (data not shown). During irradiation, the reactors were continuously stirred using a magnetic stir plate and sterile stir bars, and maintained at 22 °C in a water bath cooled by a recirculating chiller. Virus samples were exposed to simulated sunlight for up to 48 hours. At each sampling point, 1 mL sub-samples were collected and stored at -20 °C until analysis.

SI-Text-4. PNA/Pyr actinometry

The irradiation intensity of the solar simulator was quantified using *p*-nitroanisole (PNA)/pyridine (Pyr) actinometry. The actinometry solution contained 10 mM Pyr and 10 μM PNA in 5 mL of phosphate buffer (pH 7.0). During irradiation, 1 mL aliquots were collected at pre-determined time points to measure PNA concentration using high-performance liquid chromatography (HPLC) **(**Agilent Technologies Infinity 1290, Santa Clara, CA, USA**),** equipped with a ZORBAX RP Eclipse Plus C18 column (4.6 × 100 mm, 3.5 μm, Agilent Technologies, Santa Clara, CA, USA**)**. The mobile phase consisted of 10 mM phosphoric acid (50%) and acetonitrile (50%), with a flow rate of 1.0 mL/min. PNA was detected at a wavelength of 313 nm.

The total photon irradiance ($E^{0}_{p,\,total}$, in Einstein/cm^2/s) was calculated using the following expression: $E^{0}_{p,\,total} = k[\mathrm{PNA}]_0 l/[1000\phi\sum p_\lambda(1-10^{-\epsilon\lambda l[PNA]_0})\Delta\lambda]$, where k is the first-order degradation rate constant of PNA, $[\mathrm{PNA}]_0$ is the initial PNA concentration (10 μM), ε_λ is the molar absorption coefficient of PNA ($M^{-1}cm^{-1}$), l is the optical path length (3 cm), ϕ is the quantum yield of PNA photodegradation (mol/Einstein, ϕ = 0.29[Pyr]+0.00029) [3], $\Delta\lambda$ is the wavelength resolution (1 nm), and p_λ is the relative spectral photon irradiance. Photon irradiance was integrated over the 280 to 400 nm range, where the PNA absorption spectrum overlaps with the solar simulator output. The total photon irradiance measured with the actinometry method was 1.4×10^{-8} Einstein/cm^2/s, showing reasonable agreement with the radiometer-derived value of 0.8×10^{-8} Einstein/cm^2/s.

SI-Text-5. PPRI measurement

$^{\bullet}$OH. To determine the steady-state concentration of hydroxyl radicals ($^{\bullet}$OH), the formation rate of 2-hydroxyl terephthalic acid (2-HTA) from terephthalic acid (TA) was measured. In air-saturated solutions, 2-HTA is produced via hydroxylation of TA with a reported yield of 35% [4]. The steady-state $^{\bullet}$OH concentration was calculated from the 2-HTA formation rate using Eq. S1 [5].

$$\frac{d[2-HTA]}{dt} = 0.35k_{\bullet OH,TA}[TA][^{\bullet}OH],\ k_{\bullet OH,TA} = 3.3\times10^{9}\ M^{-1}s^{-1} \qquad \text{Eq. S1.}$$

Experimental solutions containing TA (10 μM) and SRNOM (10 and 15 mgC/L) were irradiated under full-spectrum simulated sunlight. Aliquots were collected at regular intervals (up to 8 h) for analysis of 2-HTA formation rate (Ms^{-1}). Quantification of 2-HTA was performed using HPLC equipped with a fluorescence detector ($\lambda_{excitation}$ = 315 nm; $\lambda_{emission}$ = 425 nm). The mobile phase consisted of 85% 10 mM phosphoric acid and 15% acetonitrile, with a flow rate of 1.0 mL/min.

$^1O_2$. The steady-state concentration of singlet oxygen ($^1O_2$) was determined by monitoring the degradation rate of a probe compound, furfuryl alcohol (FFA). Experimental solutions containing FFA (10 μM) and SRNOM (10 and 15 mgC/L) were irradiated under full-spectrum simulated sunlight. Aliquots were collected at predefined time intervals over a 2-hour period to determine the first-order degradation rate constant of FFA (k_{FFA}, s^{-1}). FFA concentrations were measured by a HPLC equipped with a diode-array detector, with detection at 217 nm. The mobile phase consisted of 80% 10 mM phosphoric acid and 20% acetonitrile, at a flow rate of 1.0 mL/min. Although FFA can react with $^{\bullet}$OH at a high rate constant of 1.5×10^{10} $M^{-1}s^{-1}$ [6], the extremely low steady-state concentration of $^{\bullet}$OH (~10^{-16} M) suggests that its contribution to FFA degradation is negligible. The steady-state $^1O_2$ concentration was calculated by dividing k_{FFA} by the second-order rate constant for the reaction of $^1O_2$ with FFA, as shown in Eq. S2.

$$[^1O_2] = k_{FFA}/k_{^1O_2,FFA} \quad (k_{^1O_2,FFA} = 1.0\times10^{8}\ M^{-1}s^{-1} \text{ at 22 °C, Appiani } et\ al.\ ^{7}) \qquad \text{Eq. S2}$$

^{3}DOM*. To determine the steady-state concentrations of triplet-excited dissolved organic matter (^{3}DOM*), the degradation rate of 2,4,6-trimethylphenol (TMP) as a probe was measured. Experimental solutions containing TMP (5 μM) and SRNOM (10 and 15 mgC/L) were irradiated under full-spectrum simulated sunlight. Solutions containing TMP and SRNOM were irradiated under full-spectrum simulated sunlight, and aliquots were collected at predefined intervals over a 2-hour period to determine the first-order degradation rate constant of TMP (k_{TMP}, s^{-1}). TMP concentrations were quantified by a HPLC equipped with a fluorescence detector ($\lambda_{excitation}$ = 225 nm; $\lambda_{emission}$ = 316 nm). The mobile phase consisted of 50% 10 mM phosphoric acid and 50% acetonitrile, with a flow rate of 1.0 mL/min. To account for potential direct photolysis, all k_{TMP} values were corrected using light screening factors derived from control experiment in phosphate buffer solution. Although TMP is known to react with both $^1O_2$ and $^{\bullet}OH$, their influence on TMP degradation is considered minimal. Based on the respective second-order rate constants (approximately 6.2×10^7 and 2.8×10^9 $M^{-1}s^{-1}$ [8,9]) and the steady-state concentrations of $^1O_2$ and $^{\bullet}OH$ in our system (~10^{-14} for $^1O_2$ and 10^{-16} M for $^{\bullet}OH$) their contributions to TMP degradation are estimated to be only ~3% and ~5%, respectively. The steady-state concentration of ^{3}DOM* was then calculated by dividing k_{TMP} by the second-order rate constant for the reaction of ^{3}DOM* with TMP, as shown in Eq. S3: $k_{^3DOM^*,TMP}$ (6.3×10^8 $M^{-1}s^{-1}$ (for SRNOM [10])).

$$[^3DOM^*]_{ss} = k_{TMP}/k_{^3DOM^*,TMP} \qquad \text{Eq.S3}$$

SI-Text-6. Nested LR-PCR and qPCR analysis

To quantify viral genomes and assess genome damage in HAdV2, a nested long-range PCR (LR-PCR) was employed to amplify 1.1-kbp fragments of the viral DNA within the hexon gene. In the second step, qPCR was performed to quantify the LR-PCR products, a combined method referred to as nested LR-PCR - qPCR analysis. Viral genome copy numbers were normalized to the human

housekeeping gene β-actin to account for differences in cell recovery across samples.

Nested LR-PCR. The primer sets and LR-PCR protocol for viral genome monitoring followed the method described by Rodríguez *et al.* [11]. Primer sequences for the human β-actin gene were adopted from the study by Vázquez-Bravo *et al.* [12]. The master mix for the nested LR-PCR consisted of the following components in a 50 μL total reaction volume: 1.25 units of GoTaq polymerase, 1X of GoTaq buffer, 2.4 mM of $MgCl_2$, 0.2 mM of each dNTP, 12 μL of 4.2 μM forward and reverse primer, and 10 μL of extracted DNA template. Primer sequences are listed in **SI Table S1**. Thermal cycling conditions were as follows: an initial denaturation at 95 °C for 2 minutes, followed by 30 cycles at 95 °C for 15 seconds, 65 °C for 20 seconds, 72 °C for 90 seconds, with a final extension at 72 °C for 5 minutes. Following amplification, the nested LR-PCR products were treated with nuclease S1 (Promega, Madison, WI, USA) to digest single-stranded nucleic acids. The resulting products were then purified using the QIAquick PCR purification kit (Qiagen, Valencia, CA, USA).

qPCR Analysis. Quantification of nested LR-PCR products and human β-actin gene were performed using qPCR. The thermal cycling conditions for the HAdV2 hexon gene product were as follows: an initial denaturation at 95 °C for 10 minutes, followed by 40 cycles of 95 °C for 15 seconds, and 60 °C for 1 minute. For amplification of the human β-actin gene, qPCR conditions included an initial denaturation at 95 °C for 2 minutes, followed by 40 cycles of 95 °C for 5 seconds, and 60 °C for 20 seconds. All qPCR assays were conducted using a Mic Real-Time PCR system (Labgene Scientific SA, Châtel-Saint-Denis, Switzerland). The nested LR-PCR products were quantified using TaqMan™ Environmental Master Mix 2.0 (Applied Biosystems™, Waltham, MA, USA), while TB Green Advantage qPCR premixes (Takara Bio Inc., Shiga Prefecture, Kusatsu City, Japan) was used for β-actin gene quantification. Calibration curves for both the HAdV2 hexon gene and human β-actin gene were generated using 10-fold serial dilutions of HAdV2 nested LR-PCR products and extracted human genomic DNA (**SI Figure S4**).

SI-Text-7. Determination of virus input dose for genome monitoring experiment

To determine the appropriate virus input dose for genome monitoring experiments, HAdV2 was inoculated into confluent A549 monolayers at concentrations ranging from 2×10^1 to 2×10^5 MPN per well (corresponding to a solution concentration of 10^1 to 10^5 MPN/mL). At 48 hours of incubation, a linear increase in hexon gene copies was observed only up to 10^3 MPN/mL per well, beyond which saturation occurred (**SI Figure S7**). In contrast, hexon gene copies measured at 0 hour of incubation increased linearly with virus spiking concentration, implying the amount of virus attached to the cells increased with input viral dose. The aim was to determine a viral input range where the virus-to-cell ratio (i.e., multiplicity of infection (MOI)) remains sufficiently low to avoid replication saturation, ensuring that host cell availability does not become a limiting factor for viral genome amplification. Based on these observations, the spiking dose of maximally 2×10^3 MPN/well was selected for subsequent experiments.

Supporting Figures

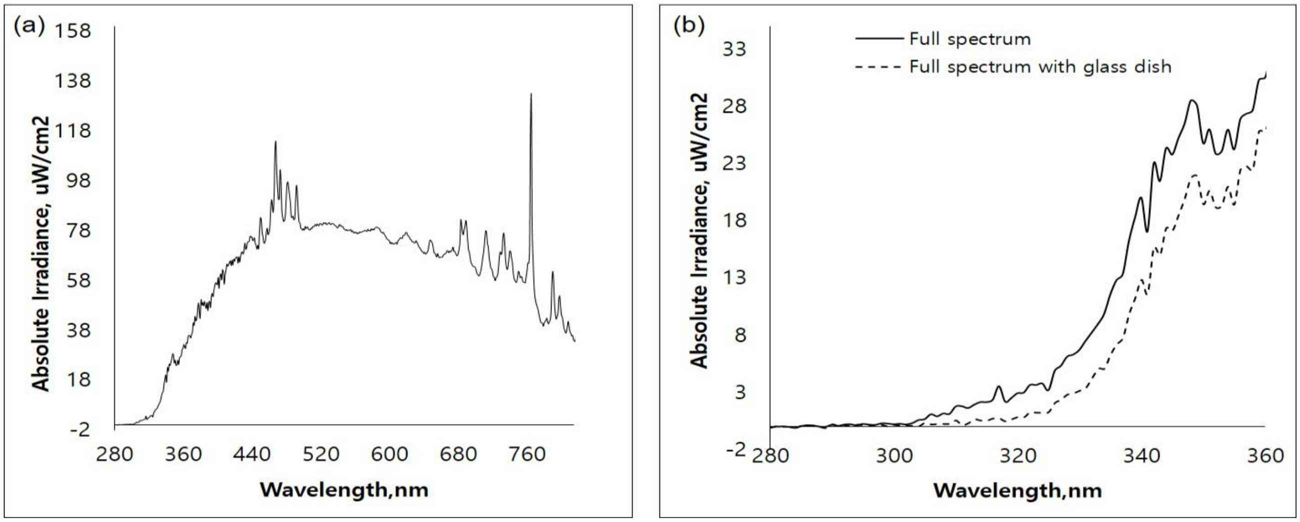

Figure S1. (a) Simulated solar spectrum generated using the AM 1.5 and atmospheric edge filter, and (b) comparison of the simulated spectrum with (dashed line) and without a glass Petri dish cover in the 280 – 360 nm range, under identical intensity (25 A) settings.

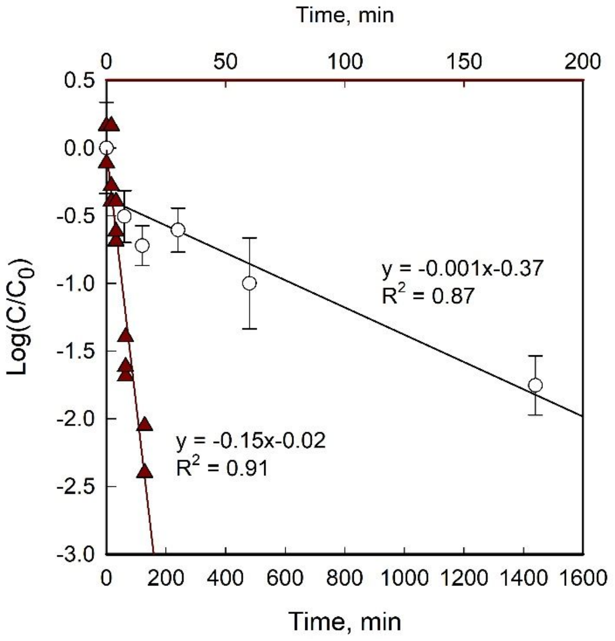

Figure S2. Solar inactivation (full spectrum, 50 A) of HAdV2 with a reactor covered by a glass Petri dish, with (red triangles, $[RB]_0 = 10\ \mu M$) and without RB (empty circles) in 10 mM phosphate buffer (pH 7.0). Red upper x-axis represents the light exposure time with RB, while black bottom x-axis represents the light exposure time without RB.

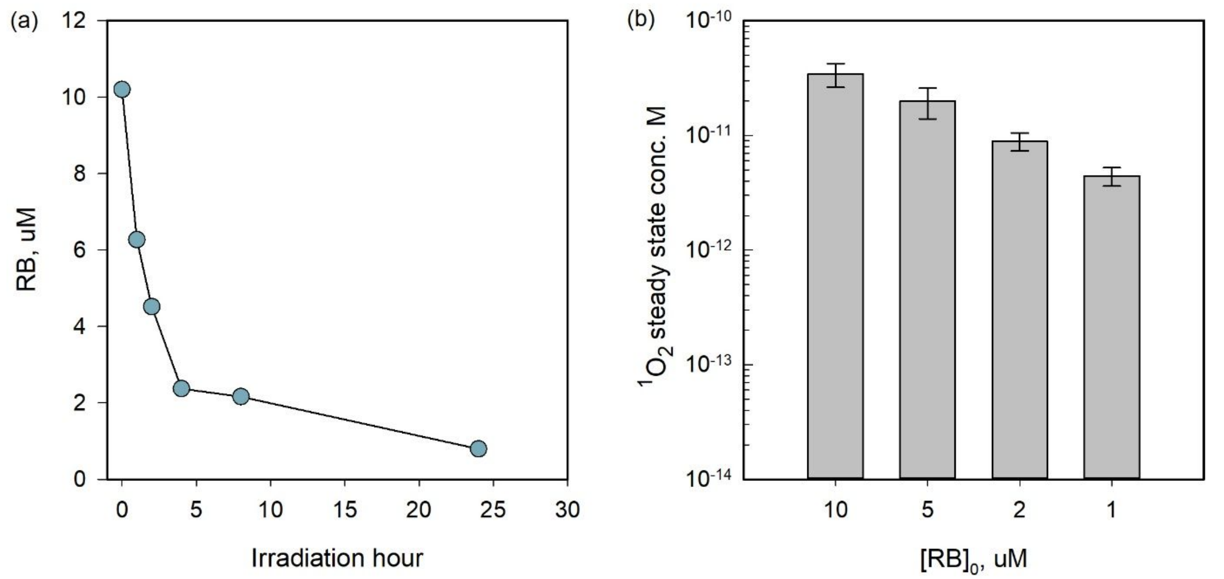

Figure S3. (a) Photo bleaching of RB ($[RB]_0$ = 10 μM) under full-spectrum simulated sunlight with a glass Petri dish cover. (b) Measured steady-state concentration of $^1O_2$ using FFA at different initial RB concentrations ($[RB]_0$ = 1-10 μM).

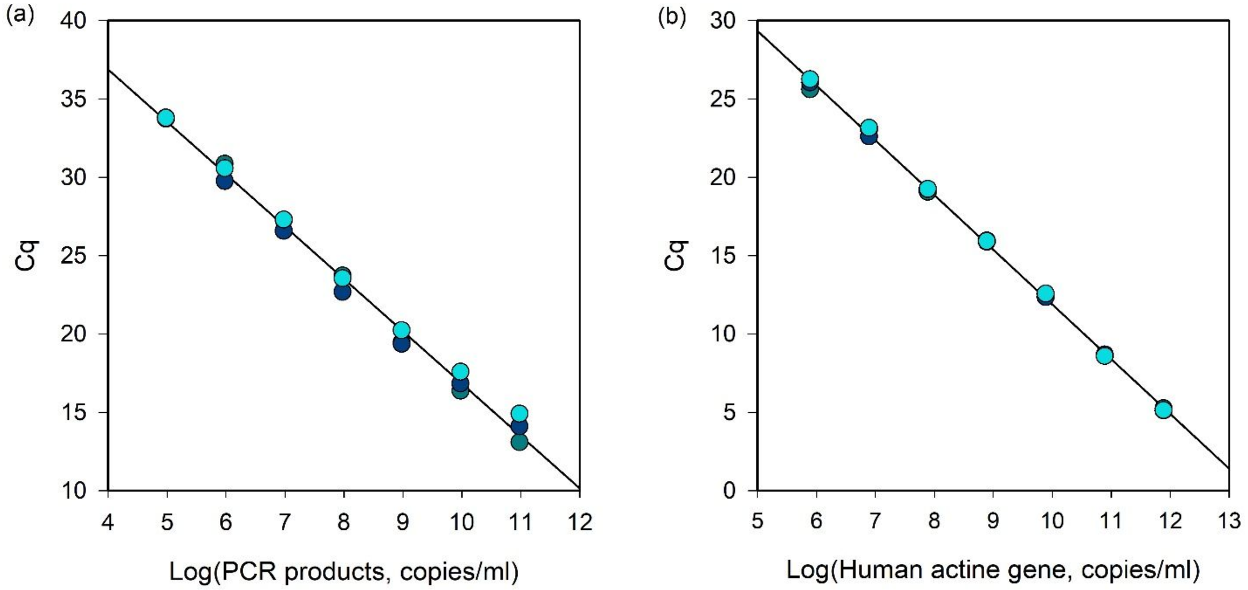

Figure S4. Standard curves of (a) HAdV2 hexon gene 1.1 kbp of the LR-PCR products targeting 177 bp amplicons and (b) human β-actin gene's 103 bp amplicons (R^2 = 0.997 and 0.999, efficiency = 105% and 91%, respectively). Each sample was analyzed in triplicate with qPCR.

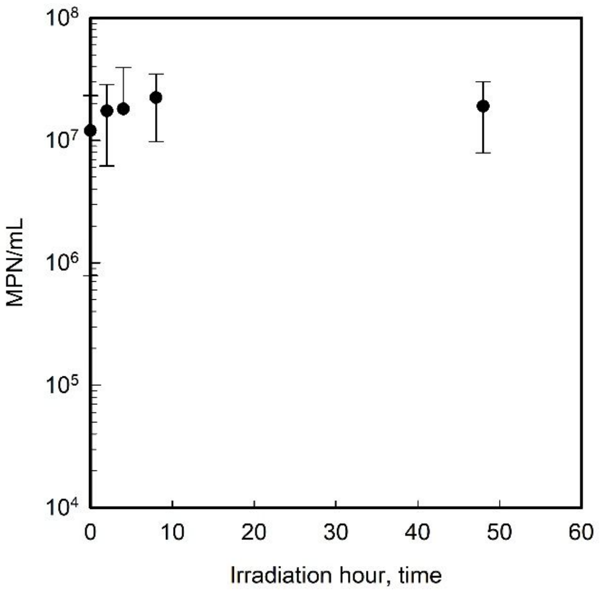

Figure S5. Dark control of HAdV2 irradiated with 15 mgC/L of SRNOM in pH 7.0 10 mM phosphate buffer up to 48 hours covered with aluminum foil. Error bars represent the standard deviations of the triplicate experiment.

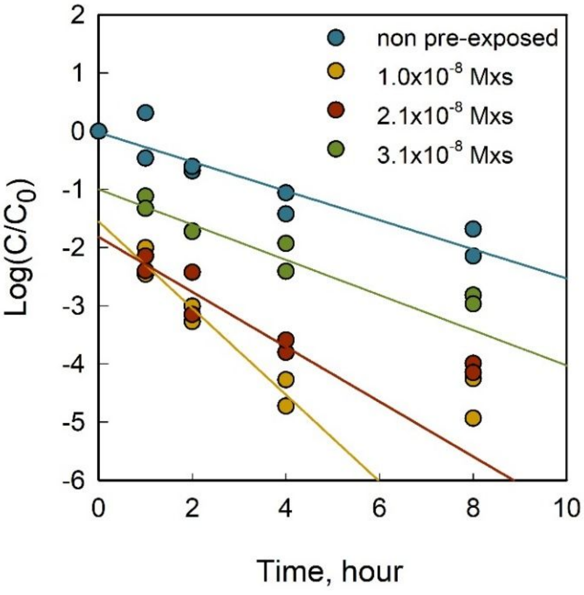

Figure S6. Solar inactivation kinetics of $^1O_2$ pre-exposed HAdV2 samples. Linear regression was performed for samples between 1 and 4 hours, except for non-pre-exposed samples. All experiments were conducted in duplicate.

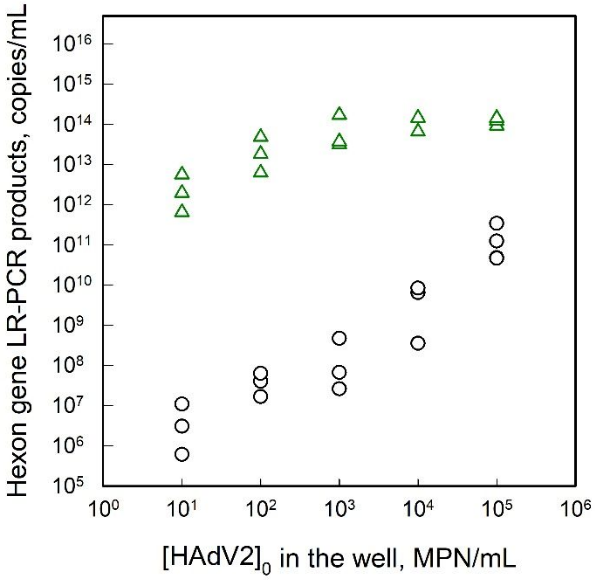

Figure S7. HAdV2 hexon gene monitoring at 0 hour (black circle) and 48 hours (green triangle) of incubation. Hexon gene was analyzed after 30 cycles of 1.1 kbp nested LR-PCR without correction by the human β-actin gene normalizer. Virus synchronization was done in triplicate and qPCR was analyzed in duplicate.

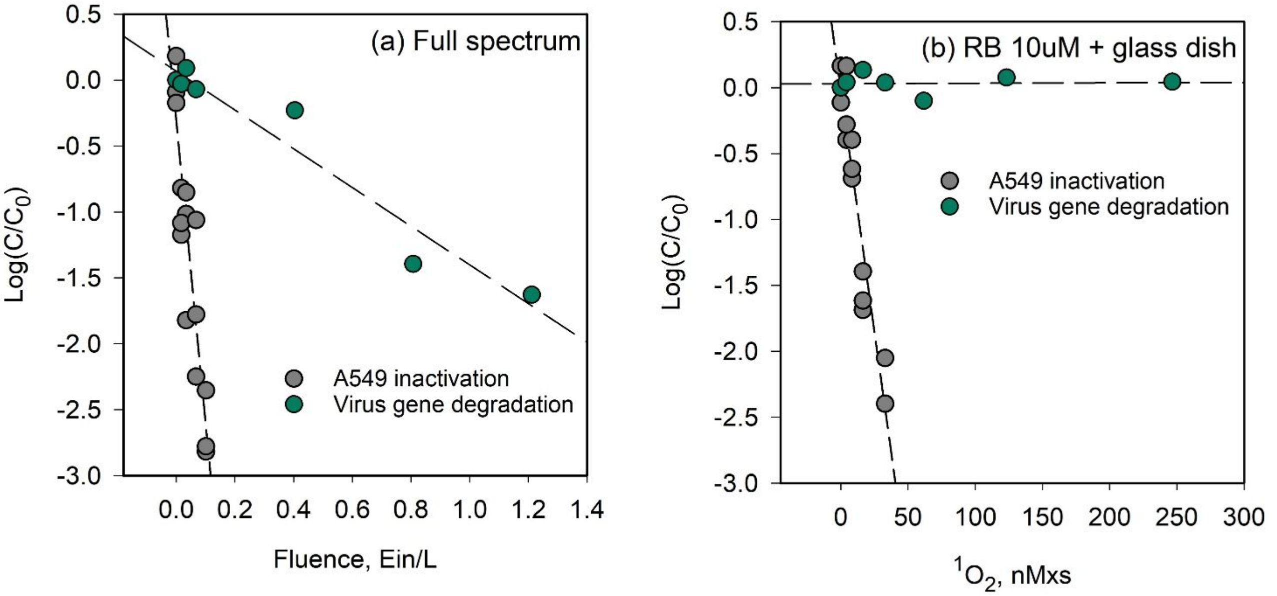

Figure S8. HAdV2 inactivation and its genome degradation under (a) direct system (full spectrum, 50 A) and (b) $^1O_2$-dominant indirect system (full spectrum, 50 A, glass Petri dish covered, 10 μM of RB). Genome degradation was measured by nested LR-PCR – qPCR method targeting the 1.1 kbp amplicon. Virus inactivation analyses were done using A549 cells with triplicate independent experiments.

Supporting Tables

Table S1. Primers and probe targeting the hexon gene of HAdV2 and β-actin gene of human genome used for nested LR-PCR and qPCR. Adapter region for HAdV2 hexon gene is highlighted with bold.

	Sequence (5'-3')	Location	size
Long range PCR Forward (HAdV2 hexon gene)	**CAC GGA GAG ATG GCT ATG CG**C GGC GGT ATC CTG CCC CTC C	17,822-18,996	1,174
Long range PCR Reverse (HAdV2 hexon gene)	CGT AGG TGC CAC CGT GGG GTT TCT AAA C		
qPCR Forward (HAdV2 hexon gene)	**CAC GGA GAG ATG GCT ATG CG**	Adapter	177
qPCR Reverse (HAdV2 hexon gene)	CAA GCG AGC GTG AGA CTC C	17,961–17,979	
qPCR probe (HAdV2 hexon gene)	FAM/ TTG CAT CCG TGG CCT TGC AGG CGC A /BHQ	17,884–17,908	
qPCR Forward (Human β-actin)	ATG AGG CTG GTG TAA AGC G	5528803-5528905	103
qPCR Reverse (Human β-actin)	AGA ACA CGG CTA AGT GTG C		

Table S2. Steady state concentrations of the PPRIs determined during simulated sunlight photolysis in the presence of SRNOM. Numbers in parentheses represent the standard error of duplicate measurements.

	$^1O_2$ (×10^{-14} M)	$^{\bullet}OH$ (×10^{-16} M)	$^3DOM^*$(×10^{-14} M)
SRNOM 10 mgC/L	4.3 (±0.4)	4.1 (±0.3)	1.1 (±0.8)
SRNOM 15 mgC/L	7.1 (±0.9)	5.6 (±0.4)	1.4 (±0.7)

Table S3. Contribution (%) of each pathway of HAdV2 solar disinfection in this study and Mattle *et al.* [13]. Mattle *et al.* evaluated HAdV2 inactivation under full-spectrum sunlight without an AE filter using water from waste stabilization ponds containing 14.4 mgC/L of organic carbon, 52 mg/L of inorganic carbon, 3.5 mg/L of nitrate, and 0.6 mg/L of nitrite. Carbonate radical ($CO_3^{\bullet -}$) was not measured (-) in this study.

	**Direct**	**$^1O_2$**	**$^{\bullet}OH$**	**$^3DOM^*$**	**$CO_3^{\bullet -}$**
SRNOM 10 mgC/L	74.2	19.8	4.5	1.5	-
SRNOM 15 mgC/L	60.7	31.7	5.9	1.7	-
Mattle *et al.* [13]	72.0	24.0	2.2	1.0	0.8

References

(1) Liu, Y.; Xu, S.; Liu, Y.; Gowda, Y. K. M.; Miao, J. Generation of Adenovirus for In Vitro and In Vivo Studies of Hepatocytes BT - Non-Alcoholic Steatohepatitis: Methods and Protocols; Sarkar, D., Ed.; Springer US: New York, NY, 2022; pp 343–358. https://doi.org/10.1007/978-1-0716-2128-8_26.

(2) Jarvis, B.; Wilrich, C.; Wilrich, P. T. Reconsideration of the Derivation of Most Probable Numbers, Their Standard Deviations, Confidence Bounds and Rarity Values. *J. Appl. Microbiol.* **2010**, *109* (5), 1660–1667. https://doi.org/10.1111/j.1365-2672.2010.04792.x.

(3) Laszakovits, J. R.; Berg, S. M.; Anderson, B. G.; O'Brien, J. E.; Wammer, K. H.; Sharpless, C. M. P-Nitroanisole/Pyridine and p-Nitroacetophenone/Pyridine Actinometers Revisited: Quantum Yield in Comparison to Ferrioxalate. *Environ. Sci. Technol. Lett.* **2017**, *4* (1), 11–14. https://doi.org/10.1021/acs.estlett.6b00422.

(4) Mark, G.; Tauber, A.; Laupert, R.; Schuchmann, H. P.; Schulz, D.; Mues, A.; Von Sonntag, C. OH-Radical Formation by Ultrasound in Aqueous Solution - Part II: Terephthalate and Fricke Dosimetry and the Influence of Various Conditions on the Sonolytic Yield. *Ultrason. Sonochem.* **1998**, *5* (2), 41–52. https://doi.org/10.1016/S1350-4177(98)00012-1.

(5) Page, S. E.; Arnold, W. A.; McNeill, K. Terephthalate as a Probe for Photochemically Generated Hydroxyl Radical. *J. Environ. Monit.* **2010**, *12* (9), 1658–1665. https://doi.org/10.1039/C0EM00160K.

(6) Minero, C.; Mariella, G.; Maurino, V.; Vione, D.; Pelizzetti, E. Photocatalytic Transformation of Organic Compounds in the Presence of Inorganic Ions. 2. Competitive Reactions of Phenol and Alcohols on a Titanium Dioxide−Fluoride System. *Langmuir* **2000**, *16* (23), 8964–8972. https://doi.org/10.1021/la0005863.

(7) Appiani, E.; Ossola, R.; Latch, D. E.; Erickson, P. R.; McNeill, K. Aqueous Singlet Oxygen Reaction Kinetics of Furfuryl Alcohol: Effect of Temperature, PH, and Salt Content. *Environ. Sci. Process. Impacts* **2017**, *19* (4), 507–516. https://doi.org/10.1039/c6em00646a.

(8) Tratnyek, P. G.; Hoigné, J. Photo-Oxidation of 2,4,6-Trimethylphenol in Aqueous Laboratory Solutions and Natural Waters: Kinetics of Reaction with Singlet Oxygen. *J. Photochem. Photobiol. A Chem.* **1994**, *84* (2), 153–160. https://doi.org/https://doi.org/10.1016/1010-6030(94)03861-9.

(9) O'Connor, M.; Helal, S. R.; Latch, D. E.; Arnold, W. A. Quantifying Photo-Production of Triplet Excited States and Singlet Oxygen from Effluent Organic Matter. *Water Res.* **2019**,

156, 23–33. https://doi.org/https://doi.org/10.1016/j.watres.2019.03.002.

(10) Erickson, P. R.; Moor, K. J.; Werner, J. J.; Latch, D. E.; Arnold, W. A.; McNeill, K. Singlet Oxygen Phosphorescence as a Probe for Triplet-State Dissolved Organic Matter Reactivity. *Environ. Sci. Technol.* **2018**, *52* (16), 9170–9178. https://doi.org/10.1021/acs.est.8b02379.

(11) Rodríguez, R. A.; Bounty, S.; Linden, K. G. Long-Range Quantitative PCR for Determining Inactivation of Adenovirus 2 by Ultraviolet Light. *J. Appl. Microbiol.* **2013**, *114* (6), 1854–1865. https://doi.org/10.1111/jam.12169.

(12) Vazquez-Bravo, B.; Gonçalves, K.; Shisler, J. L.; Mariñas, B. J. Adenovirus Replication Cycle Disruption from Exposure to Polychromatic Ultraviolet Irradiation. *Environ. Sci. Technol.* **2018**, *52* (6), 3652–3659. https://doi.org/10.1021/acs.est.7b06082.

(13) Mattle, M. J.; Vione, D.; Kohn, T.; J. Mattle, M.; Vione, D.; Kohn, T. Conceptual Model and Experimental Framework to Determine the Contributions of Direct and Indirect Photoreactions to the Solar Disinfection of MS2, PhiX174, and Adenovirus. *Environ. Sci. Technol.* **2015**, *49* (1), 334–342. https://doi.org/10.1021/es504764u.
